# Attitudes to Being Vaccinated Against COVID-19: A Survey of People With Epilepsy in China

**DOI:** 10.3389/fneur.2021.743110

**Published:** 2021-10-05

**Authors:** Shan Qiao, Ran-ran Zhang, Ting-ting Yang, Zhi-hao Wang, Xi-qin Fang, Chun-yan Fang, Jian-hong Geng, Dong-mei Zhang, Li-xin Qu, Li-li Cao, Tao Han, Xue-wu Liu

**Affiliations:** ^1^Department of Medical Genetics, School of Basic Medical Sciences, Cheeloo College of Medicine, Shandong University, Jinan, China; ^2^Department of Neurology, The First Affiliated Hospital of Shandong First Medical University and Shandong Provincial Qianfoshan Hospital, Jinan, China; ^3^Department of Neurology, Qilu Hospital, Cheeloo College of Medicine, Shandong University, Jinan, China; ^4^Department of Neurology, Zhucheng People's Hospital, Weifang, China; ^5^Department of Neurology, Affiliated Hospital of Weifang Medical College, Weifang, China; ^6^Department of Neurology, Linyi People's Hospital, Linyi, China; ^7^Department of Neurology, Dezhou People's Hospital, Dezhou, China; ^8^Department of Neurology, Shandong Provincial Hospital, Shandong University, Jinan, China; ^9^Institute of Epilepsy, Shandong University, Jinan, China

**Keywords:** COVID-19, epilepsy, vaccine, vaccine hesitation, vaccination willingness

## Abstract

**Objective:** We conducted a survey to assess vaccination coverage, vaccination willingness, and variables associated with vaccination hesitancy to provide evidence on coronavirus disease (COVID-19) vaccination strategies.

**Methods:** This anonymous questionnaire study conducted a multicenter, cross-sectional survey of outpatients and inpatients with epilepsy (PWE) registered in epilepsy clinics, in 2021, in 10 hospitals in seven cities of Shandong Province.

**Results:** A total of 600 questionnaires were distributed, and 557 valid questionnaires were returned. A total of 130 people were vaccinated against COVID-19. Among 427 unvaccinated participants, 69.32% (296/427) were willing to receive the COVID-19 vaccine in the future, and the remaining 30.68% (131/427) were unwilling to receive vaccination. Most (89.9%) of the participants believed that the role of vaccination was crucial in response to the spread of COVID-19. A significant association was found between willingness to receive the COVID-19 vaccine and the following variables: age, marital status, level of education, occupation, residence, seizure type, and seizure control after antiepileptic drug therapy. It is noteworthy that education level, living in urban areas, and seizure freedom were significantly related to willingness to receive COVID-19 vaccination.

**Conclusions:** Vaccination is a key measure for the prevention and control of COVID-19, and most PWE are willing to be vaccinated. Vaccine safety, effectiveness, and accessibility are essential in combatting vaccine hesitation and increasing vaccination rates.

## Introduction

The spread of coronavirus disease (COVID-19) caused by severe acute respiratory syndrome coronavirus 2 (SARS-CoV-2) has greatly influenced people's daily lives and health status (1, 2). The spread of infection through aerosols and contact and a lack of effective treatments may create mass panic. Vaccination seems to be an effective way to solve this problem (3). A reasonable vaccination strategy has important social significance (4, 5). To help control the COVID-19 pandemic, unprecedented efforts have been made to develop vaccines against this disease. Since January 2020, the COVID-19 viral genome has been published, and several vaccines have been authorized in China and abroad. Currently, 19 vaccines have been included in the World Health Organization (WHO) emergency use assessment list, six of which have been authorized for emergency use. Different types of COVID-19 vaccines have different protection rates, protection times, and safety levels. Owing to individual differences, there are concerns about whether people meet vaccination conditions.

Epilepsy, a common chronic neurological disease caused by abnormal brain discharge, seriously affects the physical and mental health of patients (6, 7). Both objective and subjective stress may exacerbate seizures in patients with epilepsy (PWE) (8, 9). Studies have confirmed that the stress caused by the COVID-19 outbreak may play an important role in aggravating seizures. In addition, according to the statement released by the Joint Committee on Vaccination and Immunization (JCVI) in the UK, certain underlying health conditions, including chronic neurological diseases such as epilepsy, increase the risk of morbidity and mortality from COVID-19 (10). For that reason, PWE were included in the priority vaccination group. However, both vaccination willingness and hesitancy are key factors for vaccination coverage (11, 12).

In this study, we conducted a questionnaire survey of PWE to assess vaccination coverage, vaccination willingness, and variables associated with vaccine hesitancy to provide evidence on COVID-19 vaccination strategies.

## Materials and Methods

### Study Design and Participants

The study conducted a multicenter, cross-sectional survey in June 2021, in 10 public hospitals in seven cities of Shandong Province. Shandong Province is located in eastern China and is divided into 16 cities, classified as urban and suburban districts according to population density and local economic levels. As of the 2019 census, Shandong had a population of nearly 10 million (13). Hospitals in China are divided into three levels, with level three being the highest, depending on the level of sophistication, equipment available, and staff/bed numbers. Only level two and three hospitals were included in this study.

Participants included PWE registered in epilepsy outpatient and inpatient clinics. The inclusion criteria were as follows: (1) patients diagnosed with epilepsy according to the criteria proposed by the International League Against Epilepsy (ILAE) in 2017 and (2) reasonable exclusion of other disorders. All participants were given a written description of the aims of the present study, and written consent was obtained from the participants and/or their legal guardians. Questionnaires were administered through face-to-face interviews by trained investigators. During the interviews, participants were asked to complete the questionnaire by themselves or with the help of interviewers if they had difficulty reading or writing. The survey was conducted using a self-administered, anonymous questionnaire, which consisted of six parts: (1) informed consent; (2) sociodemographic information (seven questions); (3) epilepsy-related characteristics (five questions); (4) history of COVID-19 vaccination (four questions); (5) knowledge regarding COVID-19 and its vaccine (five questions, showed in **Table 2**); and (6) attitudes toward vaccination, which included three questions: the willingness to be vaccinated, reasons to be vaccinated, and reasons for vaccine hesitation. Tick boxes were provided for PWE to respond to the inquires, which were all closed.

In this study, a total of 600 questionnaires were distributed; of which, 581 were recovered and 557 valid questionnaires were included in the data analysis (incomplete questionnaires were considered invalid). Epilepsy was diagnosed according to the ILAE 2017 definition. The type of epilepsy was classified as focal generalized, generalized, or unknown. *Seizure freedom* was defined as the “freedom from seizures for a minimum of three times the longest pre-intervention inter seizure interval (determined from seizures occurring within the past 12 months) or 12 months, whichever is longer” (14). This study was approved by the Ethics Committee of Qilu Hospital, Cheeloo College of Medicine, Shandong University, and was conducted in accordance with the Declaration of Helsinki. Written informed consent was obtained from all the study participants and/or their legal guardians.

### Statistical Analysis

Continuous variables were divided into categorical groups. All variables were shown as counts and percentages, and categorical variables were analyzed by Pearson's chi-squared test or Fisher's exact test. Variables with *p* < 0.1 in univariate analyses were subjected to multivariate logistic regression analysis with a stepwise forward elimination procedure. Multivariable logistic regression analyses were performed to examine the factors associated with PWE willingness to accept a future COVID-19 vaccine. Two-sided values of *p* < 0.05 were considered significant. Statistical analyses were performed using SPSS IBM 25.0.

## Results

### Participant Characteristics

A total of 581 PWE completed the questionnaire. Questionnaires from 24 participants were excluded from the analysis because their responses were incomplete. The basic demographic characteristics of the 557 participants are presented in Table 1. Participants' median age was 42 years [interquartile range (IQR) 30–51 years], and 51.8% were male. Among the remaining 557 participants, 130 were vaccinated against COVID-19. Among the 427 unvaccinated participants, 296 (69.32%) were willing to receive the COVID-19 vaccine in the future. Most were 18–40 years old (54.94%), married (54.58%), had a college education (22.26%), and lived in urban areas (69.48%).

**Table 1:** Characteristics of survey participants (*n* = 557).

**Demographic variables**	***n*** **(%)**	**Vaccinated (%)**	**Not yet vaccinated (%)**
**Age (years)**			
<18	67 (12.02%)	1 (0.18%)	66 (11.85%)
18–40	306 (54.94%)	81 (14.54%)	225 (40.39%)
40–60	137 (24.60%)	39 (7.00%)	98 (17.59%)
>60	47 (8.44%)	9 (1.62%)	38 (6.82%)
**Gender**			
Male	298 (53.50%)	69 (12.40%)	229 (41.11%)
Female	259 (46.50%)	61 (10.95%)	198 (35.56%)
**Marital status**			
Unmarried	224 (40.22%)	57 (10.23%)	167 (29.98%)
Married	304 (54.58%)	70 (12.57%)	234 (42.01%)
Divorced/widow	29 (5.21%)	3 (0.54%)	26 (4.67%)
**Level of education**			
University and above	124 (22.26%)	45 (8.08%)	79 (14.18%)
High school/technical secondary school	159 (28.55%)	24 (4.31%)	135 (24.24%)
Junior high school and below	274 (49.19%)	61 (10.95%)	213 (38.24%)
**Occupation**			
Professional and managerial	147 (26.39%)	49 (8.80%)	98 (17.59%)
General worker	195 (35.01%)	45 (8.08%)	150 (26.93%)
Students	53 (9.52%)	16 (2.87%)	37 (6.64%)
Farming	122 (21.90%)	9 (1.62%)	113 (20.29%)
Housewife/Retired/ Unemployed/Others	40 (7.18%)	11 (1.97%)	29 (5.21%)
**Residence**			
Urban	387 (69.48%)	88 (15.80%)	299 (53.68%)
Rural	170 (30.52%)	42 (7.54%)	128 (22.98%)
**Seizure type**			
Focal onset	367 (65.8%)	92 (16.52%)	275 (49.37%)
Generalized onset	159 (28.5%)	36 (6.46%)	123 (22.08%)
Unknown onset	31 (5.56%)	2 (0.36%)	29 (5.21%)
**Seizure control after AED therapy**			
Seizure freedom	334 (59.9%)	89 (15.98%)	245 (43.99%)
Uncontrolled	223 (40.0%)	41 (7.36%)	182 (32.68%)

### Knowledge Regarding COVID-19 Vaccine and Attitudes Toward COVID-19 Vaccination

Five knowledge questions addressed COVID-19 and its vaccine (Table 2). The first question investigated participants' perceptions of the importance of vaccination. Most (89.9%) of the participants believed that the role of vaccination was crucial in response to the spread of COVID-19. A majority (302/557) of the participants believed that there was a difference in types, safety, and effectiveness between domestic and foreign COVID-19 vaccines. The second and third questions pertained to common knowledge about COVID-19 vaccines. Although 379 (68%) participants believed that vaccination would produce long-term adverse reactions, 91 of them had been vaccinated and 117 were willing to be vaccinated in the future. A total of 503 (90.3%) participants believed that PWE could be vaccinated against COVID-19. About half of the participants (45.6%) believed that the COVID-19 vaccine would not aggravate or increase seizures; 61 of those participants were vaccinated and 114 PWE were willing to be vaccinated. Among the 131 respondents who were unwilling to accept a future COVID-19 vaccine, most participants (76.34%) believed that the role of COVID-19 vaccination in epidemic prevention and control is very important. Of those 131 respondents, 101 believed that PWE who were seizure-free could accept COVID-19 vaccination, while the remaining 30 respondents believed that PWE could not be vaccinated or were uncertain. Most (111/131) respondents who were not willing to be vaccinated or not sure believed that the COVID-19 vaccine had long-term side effects, and nearly half (40.46%) of the respondents believed that the COVID-19 vaccine might aggravate or increase seizures.

**Table 2:** Participants' knowledge regarding COVID-19 and its vaccine in relation to willingness to receive COVID-19 vaccine (*n* = 557).

**Question**	***n***	**Vaccinated (%)** **(***n*** = 130)**	**Not yet vaccinated (%) (*****n*** **= 427)** **Willingness to be vaccinated (response)**	
			**Yes (%) (*n* = 296)**	**No and not sure (%) (*n* = 131)**
**The role of the COVID-19 vaccination in epidemic prevention and control**				
Very important	501 (89.95%)	126 (22.62%)	275 (49.37%)	100 (17.95%)
Little help	13 (2.33%)	1 (0.18%)	4 (0.72%)	8 (1.44%)
Not sure	43 (7.72%)	3 (0.54%)	17 (3.05%)	23 (4.13%)
**I believed that there were differences in types, safety, and effectiveness between domestic and foreign vaccines COVID-19 vaccines**				
Yes	302 (54.22%)	53 (9.52%)	191 (34.29%)	58 (10.41%)
No	216 (38.78%)	62 (11.13%)	94 (16.88%)	60 (10.77%)
Not sure	39 (7.00%)	15 (2.69%)	9 (1.62%)	15 (2.69%)
**I believe that COVID-19 vaccines may have long-term side effects**				
Yes	379 (68.04%)	91 (16.35%)	177 (31.78%)	111 (19.93%)
No	123 (22.08%)	20 (3.59%)	95 (17.06%)	8 (1.44%)
Not sure	55 (9.87%)	19 (3.41%)	24 (4.31%)	12 (2.15%)
**I believe that patients with controlled epilepsy (seizure freedom) can be vaccinated against COVID-19**				
Yes	503 (90.31%)	126 (22.62%)	276 (49.55%)	101 (18.13%)
No	5 (0.90%)	0	1 (0.18%)	4 (0.71%)
Not sure	49 (8.80%)	4 (0.72%)	19 (3.41%)	26 (4.67%)
**I believe that COVID-19 vaccine will not aggravate or increase seizure**				
Yes	254 (45.60%)	62 (11.13%)	114 (20.47%)	78 (14.00%)
No	177 (31.78%)	27 (4.85%)	125 (22.44%)	25 (4.49%)
Not sure	126 (22.62%)	41 (7.36%)	57 (10.23%)	28 (5.03%)

### Main Reasons for Accepting COVID-19 Vaccination

**Figure 1:**
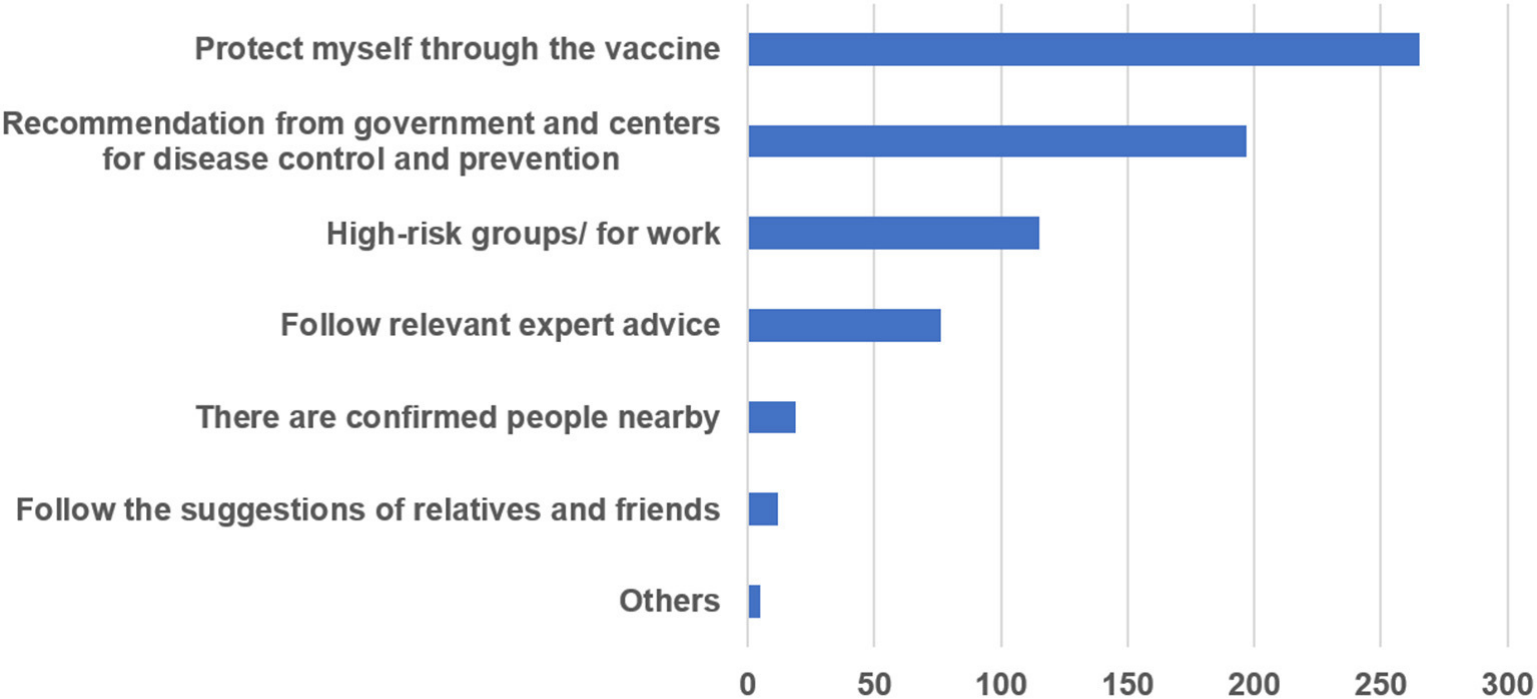
Main reasons for accepting COVID-19 vaccination.

Among participants who had not been vaccinated, 69.32% (296/427) were willing to be vaccinated. The main reasons for accepting COVID-19 vaccination were analyzed based on data from 130 respondents who had been vaccinated and 296 respondents who were willing to receive vaccinations; people were allowed to choose more than one reason. The main reason for most respondents (62.2%) was to protect themselves through vaccination. Many participants (46.24%) responded to recommendations from the government and Centers for Disease Control and Prevention. In addition, 27.0% of PWE had been vaccinated or were willing to be vaccinated because they belonged to high-risk groups or were engaged in related jobs, such as customs officers and medical workers. The other main reasons are shown in Figure 1.

### Main Reasons for COVID-19 Vaccine Hesitation

**Figure 2:**
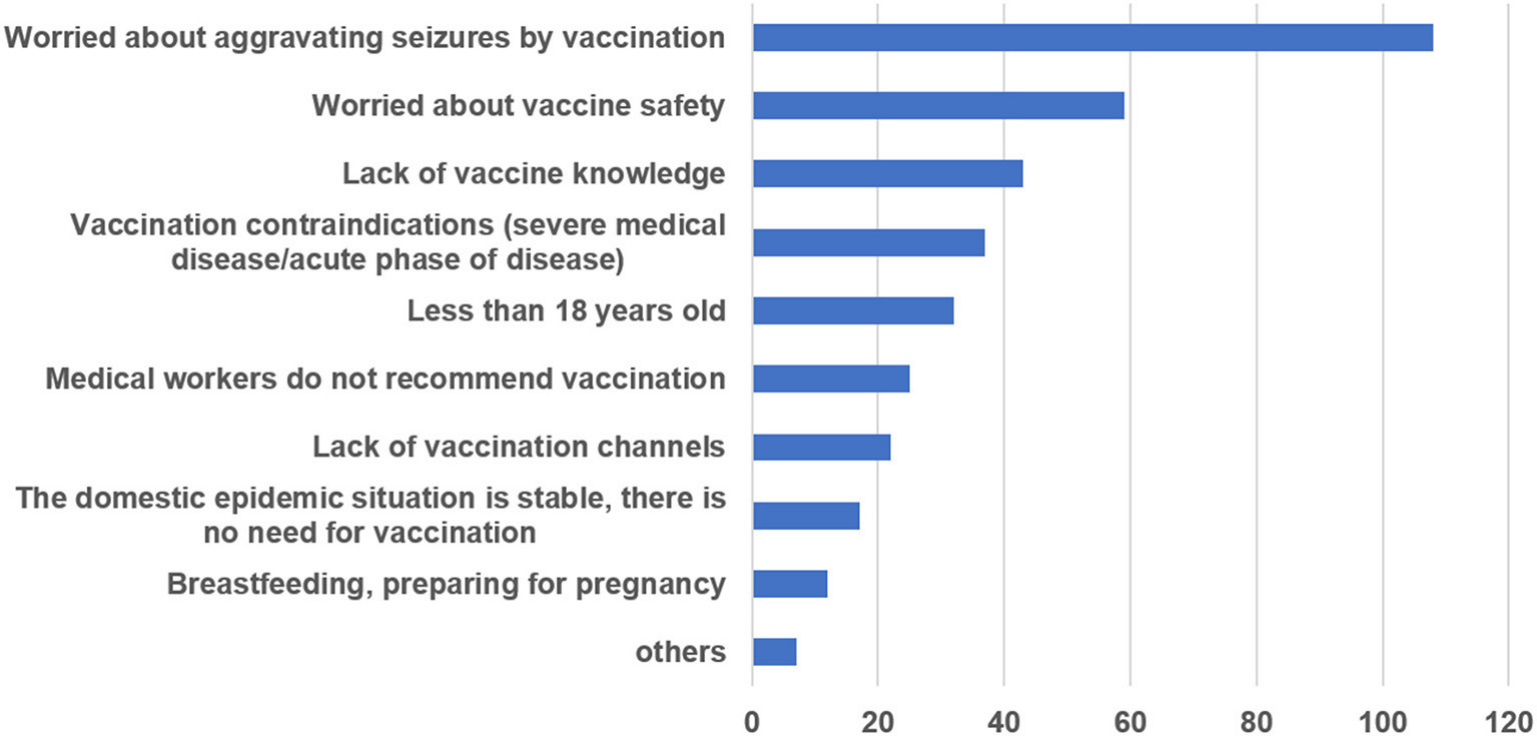
Main reasons for COVID-19 vaccination hesitation.

The main reasons for refusing or being uncertain about COVID-19 vaccination were analyzed based on data from 131 (30.68%, 131/427) respondents who were unwilling to receive vaccination; respondents could choose more than one reason for COVID-19 vaccination refusal. Most respondents (82.44%) were worried that COVID-19 vaccination might aggravate seizures, and 59 (45.04%) worried about the safety of the vaccine. Totally, 37 (28.24%) interviewees had vaccination contraindications, such as severe liver and kidney dysfunction, acute disease, or autoimmune diseases. Some participants (32.82%) did not have sufficient knowledge regarding vaccines. The results for the majority of respondents are provided in Figure 2.

### Analysis of Related Factors of COVID-19 Vaccine Hesitation

The main reasons for COVID-19 vaccine hesitation were analyzed based on data from 427 PWE who had not received vaccination. A significant association was found between the willingness to receive the COVID-19 vaccine and the following variables: age, marital status, level of education, occupation, residence, seizure type, and seizure control after antiepileptic drug (AED) therapy. No significant association was found between willingness to vaccinate and gender. The details are listed in Table 3.

**Table 3:** Sociodemographic characteristics and willingness to receive the COVID-19 vaccine among patients with epilepsy (*n* = 427).

**Predictive variables**	***n*** **(%)**	**Intent to be vaccinated**		**χ^2^-value**	***p***
		**Yes (%) (*n* = 296)**	**No and Not sure (%) (*n* = 131)**		
**Age (years)**				19.114	0.002*
<18	66 (15.46%)	34 (51.52%)	32 (48.48%)		
18–40	225 (52.69%)	173 (76.89%)	52 (23.11%)		
40–60	98 (22.95%)	66 (67.35%)	32 (32.65%)		
>60	38 (8.90%)	23 (60.53%)	15 (39.47%)		
**Gender**				0.726	0.394
Male	229 (53.63%)	154 (67.25%)	75 (32.75%)		
Female	198 (46.37%)	142 (71.72%)	56 (28.28%)		
**Marital status**				7.049	0.029*
Unmarried	167 (39.11%)	105 (62.87%)	62 (37.13%)		
Married	234 (54.80%)	169 (72.22%)	65 (27.78%)		
Divorced/widow	26 (6.09%)	22 (84.62)	4 (15.38%)		
**Level of education**				18.239	<0.01**
University and above	79 (18.50%)	68 (86.08%)	11 (13.92%)		
High school/technical secondary school	135 (31.62%)	99 (73.33%)	36 (26.67%)		
Junior high school and below	213 (49.88%)	129 (60.56%)	84 (39.44%)		
**Occupation**				126.740	<0.01**
Professional and managerial	98 (22.95%)	65 (66.33%)	33 (33.67%)		
General worker	150 (35.13%)	116 (77.33%)	34 (22.67%)		
Students	37 (8.67%)	36 (97.30%)	1 (2.70%)		
Farming	113 (26.46%)	54 (47.79%)	59 (52.21%)		
Housewife/Retired/Unemployed/Others	29 (6.79%)	25 (86.21%)	4 (13.79%)		
**Residence**				14.685	<0.01**
Urban	299 (70.02%)	224 (74.92%)	75 (25.08%)		
Rural	128 (29.98%)	72 (56.25%)	56 (43.75%)		
**Seizure type**				30.924	<0.01**
Focal onset	275 (64.40%)	216 (78.55%)	59 (21.45%)		
Generalized onset	123 (28.81%)	65 (52.85%)	58 (47.15%)		
Unknown onset	29 (6.79%)	15 (51.72%)	14 (48.28%)		
**Seizure control after AED therapy**				35.717	<0.01**
Seizure freedom	245 (57.38%)	198 (80.82%)	47 (19.18%)		
Uncontrolled	182 (42.62%)	98 (53.85%)	84 (46.15%)		

* *p < 0.05*,

** *p < 0.01*.

### Logistic Regression Analysis for Factors Associated With PWE's Willingness to Accept a Future COVID-19 Vaccine

Multivariable logistic regression analyses were performed to examine the factors associated with PWE willingness to accept a future COVID-19 vaccine. Variables with *p* < 0.1 in univariate analyses were subjected to multivariate logistic regression analysis. The likelihood ratio test was used to evaluate the regression model, and a value of *p* < 0.001 was obtained. Multivariate analysis results, summarized in Table 4, revealed three factors of PWE who were willing to receive the COVID-19 vaccine: education level [odds ratio (OR) = 2.611, *p* = 0.013, 95% confidence interval (CI) = 1.222–5.579], urban residents (OR = 3.821, *p* < 0.01, 95% CI = 2.043–7.149), and seizure freedom (OR = 0.181, *p* < 0.01, 95% CI = 0.075–0.437). Age and marital status were not statistically significant in this model (see Table 4). This prediction model passed the Hosmer–Lemeshow goodness-of-fit test (χ^2^ = 11.718, df = 8, *p* = 0.164), indicating that the model was a good fit.

**Table 4:** Logistic analysis of predictors for taking the willingness to do the COVID-19 vaccine (*n* = 427).

**Predictive variables**	***p*** **-value**	**OR**	**95% CI**
**Age (years)**	0.080	–	–
<18	0.921	0.954	0.377–2.417
18–30	0.048	0.411	0.170–0.993
31–40	0.297	0.613	0.245–1.536
41–50	0.647	0.795	0.298–2.123
51–60	0.693	1.233	0.435–3.499
**Marital status**	0.062	–	–
Unmarried	0.064	3.129	0.935–10.477
Married	0.285	1.897	0.586–6.140
**Level of education**	0.020^†^	–	–
Junior high school and below	0.013^†^	2.611	1.222–5.579
High school/technical secondary school	0.278	1.561	0.698–3.489
Resident in Urban	<0.01^††^	3.821	2.043–7.149
**Seizure type**	0.343	–	–
Focal onset	0.219	0.480	0.149–1.546
Generalized onset	0.157	0.493	0.185–1.315
Seizure freedom	<0.01^††^	0.181	0.075–0.437

† *p < 0.05*,

†† *p < 0.001; 95% CI: 95% Confidence Interval*.

## Discussion

Our study reports vaccination coverage, vaccination willingness, and variables associated with vaccination hesitancy among PWE in east China during the COVID-19 pandemic. The results showed that 23.34% of participants had been vaccinated against COVID-19. Among the interviewees who had not yet been vaccinated, most were willing to be vaccinated and believed that vaccination is an effective measure for epidemic prevention and control. Nearly one-third of respondents, however, were unwilling to be vaccinated. We analyzed the potential factors associated with PWE's willingness to accept a future COVID-19 vaccine and found that their apprehension stemmed from concern about vaccination side effects and fear of worsening epilepsy.

The ongoing COVID-19 pandemic has had a tremendous impact on public health and led to a significantly increased mortality (15). As per the WHO report of August 14, 2020, there were 20,730,456 confirmed cases of COVID-19, including 751,154 deaths worldwide (16). To decrease the spread of COVID-19, a safe and effective vaccine is the most effective measure; vaccination boosts population immunity and lowers illness severity. Based on the difference in technology routes, there are differences in in domestic and foreign vaccines. Currently, there are three approved vaccines in China, including inactivated vaccines, adenoviral carrier vaccines, and recombinant subunit vaccines (CHO cells). Countries such as the United States, the United Kingdom, Russia, and India have also used various new vaccine technologies, such as mRNA vaccines. There could be differences in various COVID-19 vaccines in terms of effectiveness or safety (17, 18). Febrile seizures are one of the most common adverse reactions of inactivated vaccine, but COVID-19 vaccine-induced febrile seizures were not reported in the phase II and the phase III clinical experiments. The subunit protein vaccine is considered one of the safest vaccines, and there is no safety data for the large-scale three-phase clinical trials reported on such vaccines at home and abroad. As for adenoviral carrier vaccines, there is no data on long-term follow-up and safety in China. However, the phase III clinical experiments of AstraZeneca vaccine based on this technology route reported a subject who experienced seizures after COVID-19 vaccination (18, 19). Currently, although there are no reports on the difference between domestic and foreign vaccines concerning safety and effectiveness and there is also no evidence that foreign and domestic vaccines differ in either effectiveness or safety, part of patients with epilepsy appear to believe such differences exist in our study. This might suggest that the importance of popularizing vaccine knowledge and the inherent concept of the subject or the way to obtain information may affect a decision to vaccination.

As of August 16, 2021, according to the official report released by the WHO, the global accumulated report of people inoculated with a new coronavirus vaccine is 4,695,164,125 (16). According to the basic propagation index value of neo zag pneumonia, 47–85% of people are required to be infected or vaccinated in the population to achieve the protection effect of population immunity. However, there are billions of people worldwide, in the short term, even in the most optimistic scenarios, vaccine production would likely be not sufficient. Therefore, there is an urgent need to increase vaccine production, develop scientific immune strategies, and promote herd immunity. With the emergence of effective COVID-19 vaccines, there remains the societal phenomenon of vaccine hesitancy, which could significantly reduce the efficacy of COVID-19 mass-vaccination programs and present a hurdle for the prevention of infectious diseases or the mitigation of new pandemics in the future. Vaccine hesitation refers to the refusal or delay of vaccination due to the public's lack of awareness of a vaccine's safety, effectiveness, and disease prevention, as well as the influence of factors such as religion, politics, and the law (19, 20). Previous studies showed that the main factors affecting vaccine hesitation included confidence in the safety and effectiveness of the vaccine, insufficient awareness of the hazards of diseases, doubts about the necessity of vaccination, vaccine availability, vaccine price acceptance, and availability of vaccination services (5, 21, 22).

Despite widespread mediatization of the successful development of COVID-19 vaccines, in our study, up to one-third of respondents who had not been vaccinated were unwilling to accept vaccination. The chief causes for their apprehension were concerns about vaccination side effects and the fear of worsening their epilepsy. These findings are consistent with those of a Lithuanian study, which found that concerns about vaccination safety and efficacy, along with fear of worsening epilepsy, were the two most common causes of vaccine hesitation among PWE (23). Our findings indicate a higher vaccine hesitancy rate than that in other studies from healthy populations in China, where up to 88.6% of all respondents would likely agree to be vaccinated against COVID-19 (24, 25). Most respondents (82.44%) who remained vaccine hesitant were concerned that COVID-19 vaccination might aggravate seizures, even though there is currently no evidence to suggest that having epilepsy is specifically associated with a higher risk of side effects from a COVID-19 vaccine (26). Finally, around a third of adult patients in our study unwilling to be vaccinated had vaccination contraindications (such as severe liver and kidney dysfunction, autoimmune diseases, or pregnancy), medical workers do not recommend vaccination, and could not get the vaccine.

Experts are gradually publishing advice for special groups including pregnant women and people with chronic diseases, which will increase their willingness to vaccinate and reduce concerns about vaccination safety. ILAE released recommendations about epilepsy and COVID-19 vaccines in February and March 2021 (27), stating that epilepsy is not a contraindication for COVID-19 vaccination; for people with epilepsy, the risk of COVID-19 infection and potential complications far outweighs the risk of side effects from a COVID-19 vaccine. As with other vaccines, however, fever can develop after a COVID-19 vaccination, which could lower the seizure threshold in some patients. Antipyretics taken regularly for 48 h after vaccination minimizes that risk, and paracetamol/acetaminophen can be used. A cross-sectional study in Kuwait on the safety and tolerability of COVID-19 vaccines among people with epilepsy showed that post-vaccination, most of their cohort (93.9%) did not experience any seizure worsening, and no patients were hospitalized because of serious side effects. In their study, only 6.1% of the vaccinated group had seizure worsening; they reported a patient who had seizure worsening experience (status epilepticus) within the last 3 months before the vaccination who had a fever for 3 days post-vaccination (28). Another study of 54 PWE in a German tertiary epilepsy center found that vaccination against COVID-19 appeared to be well-tolerated, supporting the recommendation of vaccination to PWE (29). However, more extensive and long-term studies are needed to evaluate the long-term safety of the different vaccines. A good explanation and clarification of facts to patients by providing evidence-based information on the vaccine's safety might help a PWE to make the best vaccination decision possible. So far, there has been no large-scale clinical study on the safety and effectiveness of COVID-19 vaccine among PWE reported in China. Our team has launched a prospective study on the safety and tolerability of the COVID-19 vaccine among people with epilepsy, and the results will provide accumulated data in the future.

A binary logistic regression analysis revealed that urban residents and seizure freedom greatly increased the odds ratio that the study participant would prefer vaccination. This suggests that in the PWE population, the lack of confidence in the safety of the vaccine is an important reason for vaccine hesitation, which can reduce vaccination rates. Vaccine trust is affected by many factors, such as limited data on the safety of PWE vaccination, insufficient publicity and education, and lack of knowledge about vaccination. In addition, the availability of vaccines is of great significance. Vaccine accessibility comprises vaccine prices, vaccine supplies, and vaccination services (11, 19, 20). Appointment for triage vaccination and shortage of human resources have resulted in insufficient vaccination service capacity. The shortage of vaccine supplies has also affected vaccination rates. In our study, 16.79% (22/131) of vaccine hesitators lacked routes for vaccination. Vaccine safety, effectiveness, and accessibility are key to improving vaccine hesitation and submission rate (30). Given that more than half of the PWE population will first consult a neurologist for information about the COVID-19 vaccine, the attitude of epilepsy experts on the safety and effectiveness of the vaccine is also an important factor affecting the vaccination rate of the PWE population.

This study had some limitations. Although this study is an anonymous survey, there is still a gap between it and the fact that vaccination is under real conditions (31, 32). The subjects of this study were mainly from east China and are not representative of the country as a whole. In addition, due to the lack of a control group, this sample cannot be directly compared with the general population. A relatively small sample size may have reduced the generalizability of the results. Therefore, a larger sample size and a larger coverage study are required. Given that vaccine hesitation depends to a large extent on cultural, social, and economic factors, different PWE populations may show selection bias. A larger scale and sample size study can provide data support for the safety of PWE for COVID-19 vaccination. Our team has launched a prospective study on the willingness and safety of the COVID-19 vaccination, and the research results will also provide objective data for the safety assessment of COVID-19 vaccination in PWE.

## Conclusion

Vaccination is a key measure for the prevention and control of the COVID-19 epidemic, and most PWE are willing to get vaccinated. Vaccine safety, effectiveness, and accessibility are essential in fighting vaccine hesitation and increasing vaccination rates. At present, a number of clinical studies on COVID-19 vaccines are in progress, including research on special populations such as epilepsy patients. We believe that the accumulated research results will provide more data for PWE on vaccine safety and effectiveness.

## Data Availability Statement

The original contributions presented in the study are included in the article/supplementary material, further inquiries can be directed to the corresponding author/s.

## Ethics Statement

The studies involving human participants were reviewed and approved by the Ethics Committee of Qilu Hospital, Cheeloo College of Medicine, Shandong University. Written informed consent to participate in this study was provided by the participants' legal guardian/next of kin.

## Author Contributions

X-wL conceived the study and participated in its design and coordination. SQ searched the literature and drafted the manuscript, tables, and figures. R-rZ collected and organized the data. T-tY, Z-hW, X-qF, C-yF, J-hG, D-mZ, L-xQ, L-lC, and TH assisted in collecting data. All authors contributed to the article and approved the submitted version.

## Funding

This work was supported by the National Natural Science Foundation (No. 81873786).

## Conflict of Interest

The authors declare that the research was conducted in the absence of any commercial or financial relationships that could be construed as a potential conflict of interest.

## Publisher's Note

All claims expressed in this article are solely those of the authors and do not necessarily represent those of their affiliated organizations, or those of the publisher, the editors and the reviewers. Any product that may be evaluated in this article, or claim that may be made by its manufacturer, is not guaranteed or endorsed by the publisher.
